# Data Homogeneity Effect in Deep Learning-Based Prediction of Type 1 Diabetic Retinopathy

**DOI:** 10.1155/2021/2751695

**Published:** 2021-12-28

**Authors:** Jui-En Lo, Eugene Yu-Chuan Kang, Yun-Nung Chen, Yi-Ting Hsieh, Nan-Kai Wang, Ta-Ching Chen, Kuan-Jen Chen, Wei-Chi Wu, Yih-Shiou Hwang, Fu-Sung Lo, Chi-Chun Lai

**Affiliations:** ^1^School of Medicine, National Taiwan University College of Medicine, Taipei 106, Taiwan; ^2^Department of Computer Science and Information Engineering National Taiwan University, Taipei 106, Taiwan; ^3^Department of Ophthalmology, Chang Gung Memorial Hospital, Linkou Medical Center, Taoyuan 333, Taiwan; ^4^College of Medicine, Chang Gung University, Taoyuan 333, Taiwan; ^5^Graduate Institute of Clinical Medical Sciences, Chang Gung University, Taoyuan 333, Taiwan; ^6^Department of Ophthalmology, National Taiwan University Hospital, Taipei 100, Taiwan; ^7^Department of Ophthalmology, Edward S. Harkness Eye Institute, Columbia University, New York, New York 10032, USA; ^8^Graduate Institute of Clinical Medicine, College of Medicine, National Taiwan University, Taipei 106, Taiwan; ^9^Department of Ophthalmology, Chang Gung Memorial Hospital, Xiamen 361028, China; ^10^Department of Ophthalmology, Jen-Ai Hospital Dali Branch, Taichung 400, Taiwan; ^11^Division of Pediatric Endocrinology and Genetics, Chang Gung Memorial Hospital, Linkou Medical Center, Taoyuan 333, Taiwan; ^12^Department of Ophthalmology, Chang Gung Memorial Hospital, Keelung 204, Taiwan

## Abstract

This study is aimed at evaluating a deep transfer learning-based model for identifying diabetic retinopathy (DR) that was trained using a dataset with high variability and predominant type 2 diabetes (T2D) and comparing model performance with that in patients with type 1 diabetes (T1D). The Kaggle dataset, which is a publicly available dataset, was divided into training and testing Kaggle datasets. In the comparison dataset, we collected retinal fundus images of T1D patients at Chang Gung Memorial Hospital in Taiwan from 2013 to 2020, and the images were divided into training and testing T1D datasets. The model was developed using 4 different convolutional neural networks (Inception-V3, DenseNet-121, VGG1, and Xception). The model performance in predicting DR was evaluated using testing images from each dataset, and area under the curve (AUC), sensitivity, and specificity were calculated. The model trained using the Kaggle dataset had an average (range) AUC of 0.74 (0.03) and 0.87 (0.01) in the testing Kaggle and T1D datasets, respectively. The model trained using the T1D dataset had an AUC of 0.88 (0.03), which decreased to 0.57 (0.02) in the testing Kaggle dataset. Heatmaps showed that the model focused on retinal hemorrhage, vessels, and exudation to predict DR. In wrong prediction images, artifacts and low-image quality affected model performance. The model developed with the high variability and T2D predominant dataset could be applied to T1D patients. Dataset homogeneity could affect the performance, trainability, and generalization of the model.

## 1. Introduction

Diabetic retinopathy (DR) is a severe vascular complication that may lead to blindness in patients with type 1 diabetes (T1D) [1]. As early detection and intervention can delay disease progression, patients are encouraged to undergo eye examination 3–5 years after the onset of disease and an annual DR screening thereafter [2, 3]. Despite the benefits of early treatment, approximately 60% of patients receive regular DR screening [2]. Reported reasons for nonadherence to recommended annual screening include cost, lack of access to eye care, and no perceived need [4]. Therefore, automated detection may fill these resource gaps and even improve patient outcomes by providing timely detection.

In an era of advancing technology in artificial intelligence, numerous studies have proven the effectiveness of applying deep convolutional networks for detecting DR [5–7]. However, wide variability exists in the approaches to prediction problems across different studies [8]. Understanding the factors that influence the reliability and robustness of the algorithm is important for clinical deployment and may help ensure consistency in performance under various conditions. A previous study reviewed the potential factors [9]; however, whether different etiologies of diabetes or variability of training images affect the algorithm's performance has yet to be investigated.

Diabetes patients can be classified into two major categories based on different etiologies: T1D, which is caused by insulin deficiency and is also known as insulin-dependent diabetes, and type 2 diabetes (T2D), which is caused by insulin resistance and is also known as insulin-independent diabetes mellitus. T2D accounts for most cases of diabetes. In the US population, >90% of patients with diabetes have T2D, whereas T1D accounts for only 5% [10]. T1D predominantly affects the European population [11], and its prevalence in the Asian population is even lower; for example, T1D is present in <1% of the diabetic population in Taiwan [12]. Although T1D presents as the minority in the diabetic population, patients with T1D are more likely to develop DR and have more severe visual outcomes than patients with T2D [1, 2, 13, 14]. A study reported that youth with T1D also develop DR faster than those with T2D [15]. When evaluating the cause of vision impairment in diabetes, DR accounts for 86% of poor visual acuity in T1D and only 33% in T2D [14]. As machine learning has been widely used for the automatic detection of DR, most images are obtained from the dataset predominantly containing the images of T2D patients. Furthermore, the investigation of the performance of models in identifying DR in the specific T1D population is limited.

To assess whether different etiologies of diabetes (i.e., T1D and T2D) affect the performance and robustness of deep learning models, we conducted this study using deep learning models trained using two datasets: one from open-access datasets with high image variability from T2D patients predominantly and the other one consisting of images obtained only from T1D patients followed at a single medical center. As our dataset is small compared with the recommended size [9], the deep transfer learning method is preferably used, which allows for low training cost and the use of a smaller training dataset by reusing a pretrained network to solve a different task [16]. The performance and heatmaps of deep learning models trained with the two datasets were then compared.

## 2. Materials and Methods

### 2.1. Datasets

An open-access dataset was subsampled from one of the Kaggle datasets, namely, Train.001, which is a publicly available dataset provided by EyePACS [17], which contained a group of patients with a mean age of around 55.4 years and a standard deviation of 11.3 years [5]. The other dataset of retinal fundus images was retrospectively acquired from T1D patients at a 3700-bed medical center, Chang Gung Memorial Hospital, Linkou Medical Center, Taiwan, between 2013 and 2020. All T1D patients were from the Chang Gung Juvenile Diabetes Eye Study [18, 19] and diagnosed based on the World Health Organization diagnosis criteria [20]. The T1D dataset consisted of patients with a mean age of 25.7 years and a standard deviation of 5.8 years. In the T1D dataset, two types of color fundus cameras were used (Topcon Medical Systems, Oakland, NJ, USA; Kowa, Tokyo, Japan, and Digital Non-Mydriatic Retinal Camera, Canon, Tokyo, Japan). Image resolution in both datasets ranged from 1,000 × 1,500 to 2,500 × 3,500 pixels. This study was approved by the Institutional Review Board of Chang Gung Memorial Hospital (no. 201900477B0) and adhered to the tenets of the Declaration of Helsinki.

### 2.2. Classification of DR

Retinal fundus images from T1D patients were graded by two trained retinal ophthalmologists (EYK and NKW) according to the International Clinical Diabetic Retinopathy Disease Severity Scale. Images with artifacts, shadows, or poor quality that could not be classified were excluded. Retinal ophthalmologists were unaware of clinical information, such as demographics, laboratory data, and prior treatment. On the other hand, DR classification in the Kaggle dataset was defined according to the labels provided with the dataset. In this study, DR was defined as the diagnosis of DR at any stage [21].

### 2.3. Data Preprocessing and Division

**Figure 1:**
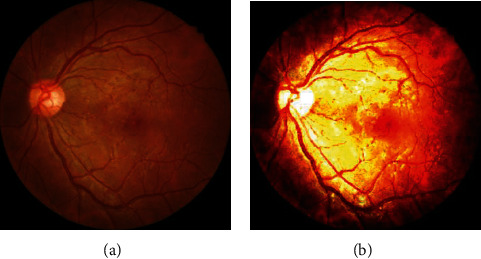
Fundus image after (a) cropping and (b) normalization.

**Figure 2:**
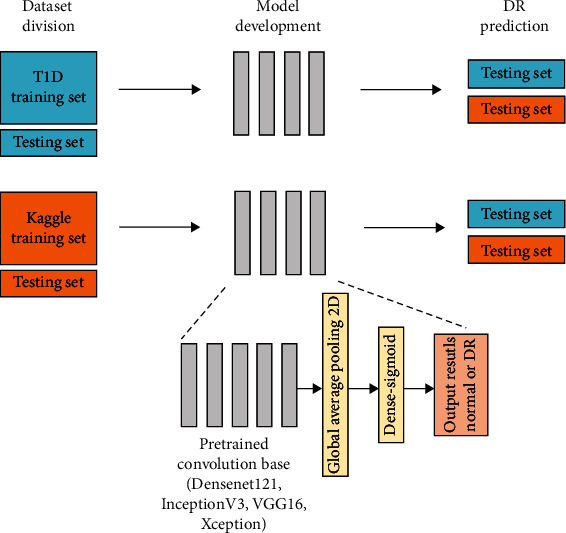
Schematic of the development and evaluation of models. Two groups of models were trained using the T1D and Kaggle training sets, and they were tested with both the T1D and Kaggle testing sets.

All input images from both datasets were cropped and resized using OpenCV-python to a 320-pixel wide square that tightly contained the circular fundus region. Monochromatic fundus photography and images not having both the optic disc and macular region were filtered out (Figure 1). For each dataset, images were randomly divided into two sets: two-thirds in the training set to develop the model and one-third in the testing set to evaluate model performance (Figure 2). Then, the training set was further divided into two subsets: two-thirds of the training set for optimizing the weights of the network and one-third as the validation set to select hyperparameters for the model. As images from the T1D dataset may come from the same patients, to avoid data leakage, images from same patients were placed in the same sets. After division, the images were randomly shuffled in their own dataset to reduce overfitting and variance before training and were then further batch normalized by subtracting the average and dividing by the standard deviation calculated from the training dataset using ImageDataGenerator of Keras API. Real-time data augmentation was applied by randomly rotating, shifting, and shearing the images during the model training based on previously published methods [22].

### 2.4. Architecture and Evaluation of the Model

The deep transfer learning model consisted of a pretrained convolutional neural network (CNN), followed by a global average pooling layer and a dense layer to output prediction results (Figure 2). The weights from the pretrained model were trainable and were used to extract image features, and predictions were then made using the final classifier. Class imbalance was addressed by estimating reweighting loss. Early stopping after 8–12 epochs of no improvement was applied to avoid overfitting, and the learning curves of both the training and validation sets were plotted to detect underfitting or overfitting. Binary cross entropy was used as the loss function, and stochastic gradient descent [23] or the Adam optimizer [24] was used with a learning rate of 1e-3 to 1e-4. Hyperparameters were optimized using random search. The development and analysis of the models were implemented using Keras 2.4.3 and Tensorflow 2.4.1 on Google colaboratory [25], whereas a part of image preprocessing and gradient-weighted class activation (Grad-CAM) visualization were run in Jupyter Notebook [26]. Two groups of models trained using the T1D and Kaggle training sets were tested in both the T1D and Kaggle testing sets (Figure 2).

In our model, CNNs including Inception-V3 [27], DenseNet-121 [28], VGG16 [29], and Xception [30] were selected for model training because of their high performance in ImageNet Large Scale Visual Recognition Challenge and wide implementation in other medical image classifications [9]. All networks were pretrained on ImageNet [31]. Performance of the model with each CNN was evaluated.

### 2.5. Visualization Method

To observe how the models led to the prediction, the final convolutional layer of each model was extracted to obtain the activation map using the Grad-CAM visualization method [32], which highlighted the regions that provided an important contribution to the prediction. The activation map was then superimposed on the original image for interpretation.

### 2.6. Statistical Analysis

Receiver operating characteristic (ROC) curves were plotted using Matplotlib 3.2.2. Area under the ROC curve (AUC), sensitivity, and specificity were calculated to compare the performance of different models trained with both datasets using Python 3.7.1 and Sklearn 0.22.2. Optimal threshold of ROCs was chosen by maximizing the geometric mean of sensitivity and specificity. The descriptive results in this study are expressed as numbers and percentages for discrete variables.

## 3. Results

### 3.1. Image Characteristics

In the Kaggle dataset, 8,408 images were subsampled from the original dataset, with 6,150 (73%) images classified as normal and 2,258 (27%) images as DR. In the T1D dataset, 7,064 images from 475 patients with T1D were collected. Of these, 873 (13%) images from 79 (17%) patients were classified as DR.

### 3.2. Model Performance

**Figure 3:**
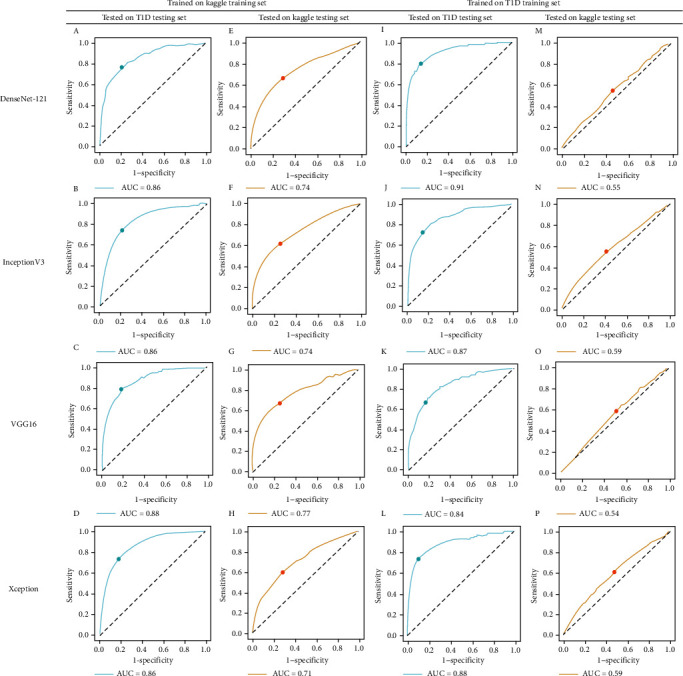
Receiver operating characteristic (ROC) curves of different transfer learning models in predicting diabetic retinopathy. The ROC curve of models that were tested with the type 1 diabetes (T1D) testing set was plotted in blue, whereas those tested with the Kaggle testing set were plotted in orange. The point on the ROC curve was the selected threshold. (e)–(h) There was a significant decrease in AUC when models previously trained with the T1D training set were tested with the Kaggle dataset. (i)–(l) The models that were previously trained with the Kaggle training set have a more robust performance when tested with the T1D testing set.

Model performance is shown in Table 1. When the models were trained using the Kaggle imaging dataset, the overall AUC reached a mean (range) of 0.74 (0.03) in the Kaggle testing set, with VGG16 providing the best performance (AUC = 0.77). AUCs increased to a mean (range) of 0.87 (0.01) when the models trained with the Kaggle training set were tested using the T1D testing set. On the other hand, the transfer learning models achieved a mean (range) AUC of 0.88 (0.03) when trained and tested using the T1D imaging dataset, with DenseNet-121 providing the best performance (AUC = 0.91) and VGG16 the worst (AUC = 0.84). However, when models that were previously trained using the T1D training set were tested using the Kaggle dataset, AUCs significantly decreased to a mean (range) of 0.57 (0.02). The corresponding ROC curves are illustrated in Figure 3.

### 3.3. Class Activation Maps

**Figure 4:**
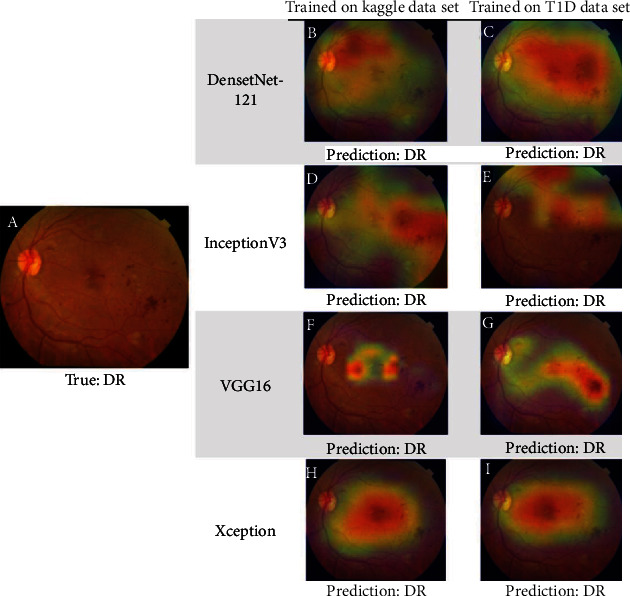
The images demonstrate the original (a) and superimposed Grad-CAM activation maps ((b)–(i)) of the selected diabetic retinopathy (DR) color fundus image. All models gave a true-positive prediction. There were some similarities in activation maps even in different transfer learning models trained with different datasets.

**Figure 5:**
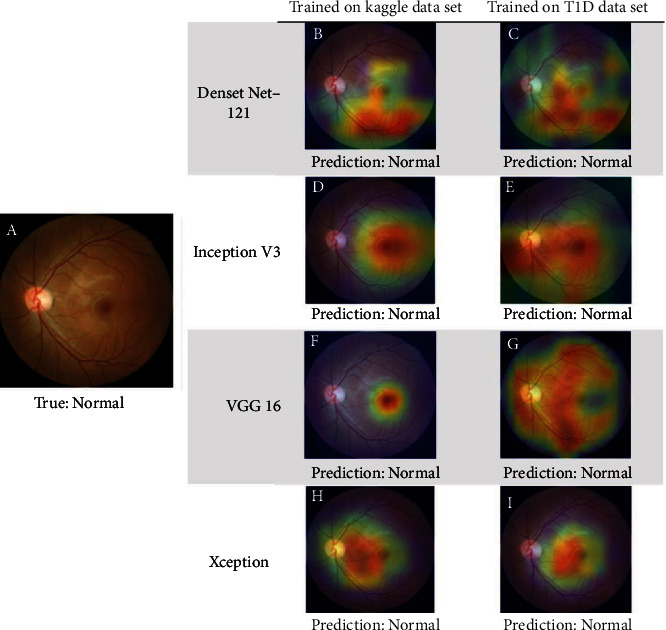
The images present the original (a) and superimposed Grad-CAM activation maps ((b)–(i)) of the selected normal color fundus image. All models gave a true-negative prediction. There was a high variation in the activation map when given a normal fundus image. Some models focus on the optic disc ((e) and (g)), whereas others highlight the retinal vessels ((b), (c), and (g)), or macular region ((d), (h), (f), and (i)).

The results of activation maps from different transfer learning models of both DR and normal cases are presented in Figures 4 and 5. Aside from highlighting the clinically observable retinal abnormalities, which were the traditional characteristic findings of DR, including microaneurysms, hemorrhages, and exudates (Figures 4(c), 4(d), and 4(g)), other regions including the macula (Figures 4(h), 4(i), 5(d), 5(h), 5(f), and 5(i)), optic disc (Figures 5(e) and 5(g)), and retinal vessels (Figures 5(b), 5(c), and 5(g)) were also occasionally highlighted. Greater similarities were observed in activation maps among transfer learning models trained using the DR fundus image (Figure 4) rather than the normal fundus image (Figure 5).

## 4. Discussion

### 4.1. Main Findings of the Present Study

In our study, we trained models using the open-access Kaggle dataset, which has high image variability and theoretically predominant T2D patients, and the T1D dataset from the single medical center. We found that the model trained using the Kaggle dataset had an average AUC of 0.74 when testing using the same dataset, but this increased to 0.87 when testing using the T1D dataset. By contrast, the model trained using the T1D dataset had high accuracy (up to AUC of 0.91) in T1D patients, but it decreased (lowest AUC of 0.54) with the Kaggle dataset. Heatmaps demonstrated weighted features of retinal microaneurysm, hemorrhage, exudation, and vessels. Dataset homogeneity dataset may affect the trainability and generalization of the model.

### 4.2. Importance of External Validation and Standardization of Hyperparameters

Previous studies proposed numerous models that achieved high performance in diagnosing DR, even when trained with only a small dataset containing thousands of images [16]. The performance of our results yielded comparable results with the previous study (AUCs ranged from 0.65 to 0.86) when using a similar data size from the Kaggle dataset [33]. However, a large DR screening validation study found that most algorithms had significant performance differences and even obtained concerning results when evaluated through external validation, even though these algorithms were already in active use in real-world clinical settings [34]. In our study, models trained using the T1D dataset also exhibited acceptable performance (AUCs between 0.84 and 0.91) when internally validated, but their performance significantly decreased when evaluated using the external dataset. These results highlight the need for rigorous training and testing of models by using datasets containing a similar distribution of target population to avoid the huge discrepancy between expected and real performance. In addition, to produce a stable and reproducible prediction outcome, considerably more hyperparameters should be standardized. Although a previous study had already investigated the large number of possible factors that influence the performance of deep learning model [9], we anticipate that many more elements still need to be determined. For instance, the etiology of DM, age range, and comorbid eye diseases were shown to be possible influencing factors in our study.

### 4.3. Different Performance Levels in Different Datasets

**Figure 6:**
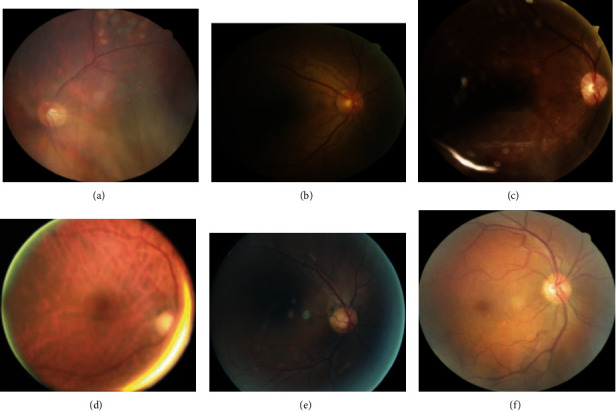
Images in the Kaggle dataset with wrong prediction. (a) False-negative in an image with foggy view and retinal laser scar. (b) False-negative in an image with poor illumination. (c) False-negative in an image with reflective spots and shadows. (d) False-positive in an image with overexposure and halo. (e) False-positive in an image with underexposure and halo. (f) False-positive in an image with exudates caused by age-related macular degeneration.

The models trained using the T1D dataset had poor performance when tested using the Kaggle dataset, whereas those trained using the Kaggle dataset showed better performance when tested using the T1D dataset. There are several possible explanations for the differences in the generalization of models trained using different datasets despite the use of the same method, similar dataset size, and imbalance ratio. When reviewing the wrong prediction images, we found that the images had similar problems affecting model prediction (Figure 6). Images from the T1D dataset were evaluated by retinal ophthalmologists, and images with poor quality or those that could not be graded were excluded. As the T1D dataset contained images of patients over multiple visits, the number of unique patients with T1D may be less than that in the Kaggle dataset, resulting in homogeneous data and thus rendering the T1D database much easier to predict for models trained using either of the databases. By contrast, images from the Kaggle dataset may not be cleaned and may contain more noise and artifacts, including out-of-focus, overexposure, or underexposure images, than images from the T1D dataset. Furthermore, the Kaggle dataset could be collected from a more diverse population with older age and higher age variation, thus, having more heterogeneous characteristics, whereas T1D patients had similar demographics and younger age. Therefore, patients in the Kaggle dataset may have other ocular diseases related to aging or other comorbidities such as cataract and age-related macular degeneration. Retinal features of ocular diseases other than DR, such as retinal exudates in age-related macular degeneration, may affect model prediction. In addition, cataracts may affect image quality. These findings have been reported in our previous studies [35, 36]. As the T1D dataset contained images of patients over multiple visits, the characteristic variation in T1D may be less than that in the Kaggle dataset, also resulting in homogeneous data. Therefore, a homogeneous dataset may have resulted in higher trainability and lower generalization of models and vice versa in a more heterogenous dataset. Therefore, heterogeneity of the testing population also influences the performance of prediction models.

### 4.4. Highlighted Regions by Grad-CAM

Typical characteristic findings of DR, such as retinal microaneurysms, hemorrhages, exudates, and neovascularization, were among the most common highlighted regions by Grad-CAM in our study, consistent with a previous report [37]. In addition, nontraditional regions including the macula and optic disc were occasionally highlighted. As DR may also present with diabetic macular edema and neovascularization of the disc, abnormal features in these regions may also be extracted. Although neurodegeneration precedes vascular lesion in DR [38], whether deep learning models can detect abnormalities before the appearance of clinically observable lesions requires further investigation.

### 4.5. Limitations

This study has several limitations. First, we only assessed how training using only the T1D dataset affects detection performance; these results may not apply to other etiologies, such as the inherited form, maturity onset diabetes of the young, or other secondary causes. Second, our T1D data were collected from a single medical center and from a single ethnicity, making the dataset relatively small with less heterogeneity. Third, DR was identified using macula-centered retinal fundus images in the T1D dataset, instead of images obtained through 7-field retinal fundus photography, as suggested by the Early Treatment Diabetic Retinopathy Study [39]. In addition, we did not justify further DR grading, which may help with the determination of treatment-required DR, because the detection of early DR in the T1D population could provide more information in patient care and education [18]. Finally, our models were developed with a limited combination of hyperparameters, and we did not conduct a combined model training on both datasets. A different implementation may thus provide different results.

## 5. Conclusion

Our study investigated a deep learning-based DR prediction model using two datasets. Our results showed that dataset homogeneity can have a significant effect on the trainability and generalization of the model. This implied that deep learning models should be trained with data similar to the target population and updated according to the landscape of DM to ensure a robust prediction and outcome. As the prevalence of diabetes continues to rise [2], along with an alarming increase in the frequency of T2D among youth [40], the epidemiology of diabetes will continue to change. In addition, activation maps produced inferred that in addition to characteristic findings of DR, the macula and optic disc may also contribute to the detection of abnormalities in fundus imaging.

## Figures and Tables

**Table 1:** Summary of the prediction performance of different transfer learning models in predicting diabetic retinopathy.

	Trained on Kaggle training set						Trained on T1D training set					
	Tested on T1D testing set			Tested on Kaggle testing set			Tested on T1D testing set			Tested on Kaggle testing set		
	AUC	SEN	SPE	AUC	SEN	SPE	AUC	SEN	SPE	AUC	SEN	SPE
DenseNet-121	0.86	0.77	0.79	0.74	0.67	0.71	0.91	0.81	0.86	0.55	0.55	0.54
InceptionV3	0.86	0.74	0.79	0.74	0.62	0.74	0.87	0.73	0.86	0.59	0.56	0.59
VGG16	0.88	0.78	0.82	0.77	0.66	0.75	0.84	0.67	0.84	0.54	0.59	0.49
Xception	0.86	0.74	0.82	0.71	0.60	0.72	0.88	0.74	0.90	0.59	0.61	0.52

T1D: type 1 diabetes; AUC: area under the curve; SEN: sensitivity; SPE: specificity.

## Data Availability

The T1D dataset is not publicly available due to the data security policy of Chang Gung Memorial Hospital and is available upon reasonable request.
